# Data regarding particle size distribution, thermal properties and gaseous phase hydration of co-milled solid dispersions composed of tadalafil and Soluplus

**DOI:** 10.1016/j.dib.2022.108451

**Published:** 2022-07-08

**Authors:** Karol Kubat, Anna Krupa, Witold Brniak, Agnieszka Węgrzyn, Dorota Majda, Agata Bogdał, Hubert Harańczyk

**Affiliations:** a Institute of Physics, Jagiellonian University, 11 Prof. S. Łojasiewicza Street, Cracow 30-348, Poland; b Faculty of Pharmacy, Department of Pharmaceutical Technology and Biopharmaceutics, Jagiellonian University Collegium Medicum, 9 Medyczna Street, Cracow 30-688, Poland; c Faculty of Chemistry, Jagiellonian University, 2 Gronostajowa Street, Cracow 30-387, Poland

**Keywords:** Processing, High-energy ball milling, Water vapour sorption, Micronization

## Abstract

A mechanical activation of the solid particles upon high-energy ball milling may considerably change the physicochemical properties of pharmaceutical compounds, including the morphology, particle size distribution, thermal properties, and surface interactions with water vapour upon gaseous phase hydration. Assessment of these changes is crucial for optimizing the manufacturing process of enabling drug products. In this article, we provide a detailed characterization of binary co-milled solid dispersions composed of tadalafil and Soluplus using a laser diffraction method, differential scanning calorimetry (DSC), gravimetric measurements and solid state ^1^H- NMR spectroscopy. The data presented in this article is directly related to our previously published research article. They complement information on the impact that both formulation and process variables may have on the properties of these binary powder formulations.

**Specifications Table**

**Table:** 

Subject	Biophysics, Materials Physics
Specific subject area	Characterization of a binary co-milled solid dispersion made of poorly soluble drug-tadalafil and an amphiphilic matrix-forming polymer-Soluplus
Type of data	Figures
How the data were acquired	Particle size distribution (PSD) - a laser diffraction method. A Mastersizer 3000 (Malvern Instruments Ltd., United Kingdom) equipped with a Hydro EV semi-automated wet sample dispersion unit and a Hydro Sight dynamic imaging accessory was used. Data was collected using Mastersizer 3000 v.3.60 software. Data was exported into ASCII files (*PSD*.csv).Thermal properties - a differential scanning calorimetry (DSC). Heat flow curves were recorded using a Mettler Toledo 821e differential scanning calorimeter (Switzerland) with the software STARe v.11.0. Data was exported into ASCII files (DSC.txt).Water vapour sorption, an ^1^H- NMR spectroscopy in the solid state. Spectra were acquired using a Bruker Avance III 300 spectrometer. Data was imported into a project of an OriginLab v. 2021b software through ONMR extension for presentations and analyses. Data was exported into ASCII files (200_60_Raw.txt, 200_60_Summary.txt)
Data format	RawAnalyzed
Description of data collection	Tadalafil was ball milled at room temperature for 1 h with Soluplus loaded in three weight ratios: 90 % (1+9), 50 % (1+1), 10 % (9+1). The first number in the mixing ratio (given in brackets) corresponds to tadalafl load while the second number corresponds to Soluplus load. The rotational speed of a solar disc was kept at three levels, i.e. 100, 200, 400 rpm. The impact of both formulation and process variables on particle size distribution (PSD), thermal properties and gaseous phase hydration of these solid dispersions was investigated using laser diffraction, DSC, and solid-state ^1^H NMR respectively.
Data source location	• Institution: Jagiellonian University in Krakow• City/Town/Region: Krakow/Małopolska• Country: Poland• Latitude and longitude and GPS coordinates: 50°03′23.40″ N 19°55′34.79″ E
Data accessibility	Repository name: Mendeley Data Data identification number: 10.17632/RNWRYSDKFG.2 Direct URL to data: https://doi.org/10.17632/RNWRYSDKFG.2 Tadalafil_Soluplus_Data - Mendeley Data [1]
Related research article	K. Kubat, A. Krupa, W. Brniak, A. Węgrzyn D. Majda, A. Bogdał, H. HarańczykHow the rotational speed of the planetary ball mill and the polymer load can influence the performance and water vapor sorption in solid dispersions composed of tadalafil and Soluplus,Particuology 2023, vol. 73, pp. 37-46.https://doi.org/10.1016/j.partic.2022.04.003.

**Value of the Data**

• Data recorded during PSD, DSC and solid state ^1^H NMR measurements allow to understand the effect of both formulation and process parameters on the morphology, molecular configuration, and water sorption of ball milled solid dispersions.
• The frequency distribution curves provide detailed information on the homogeneity of the solid dispersion.
• DSC studies are a useful tool to establish molecular interactions between the drug and the polymer induced by high-energy ball milling.
• Analysis of solid state ^1^H NMR spectra recorded for gaseous phase hydrated samples enables to predict the stability of solid dispersions upon storage.
• These data can be useful for the optimization of the ball milling process which aims in solid dispersion manufacturing, especially when bulk compounds differ significantly in particle size or the matrix-forming excipient is hygroscopic, e.g. Soluplus.
• They could facilitate a proper selection of package materials or storage conditions for solid dispersions.

## 1 Data Description

**Fig. 1:**
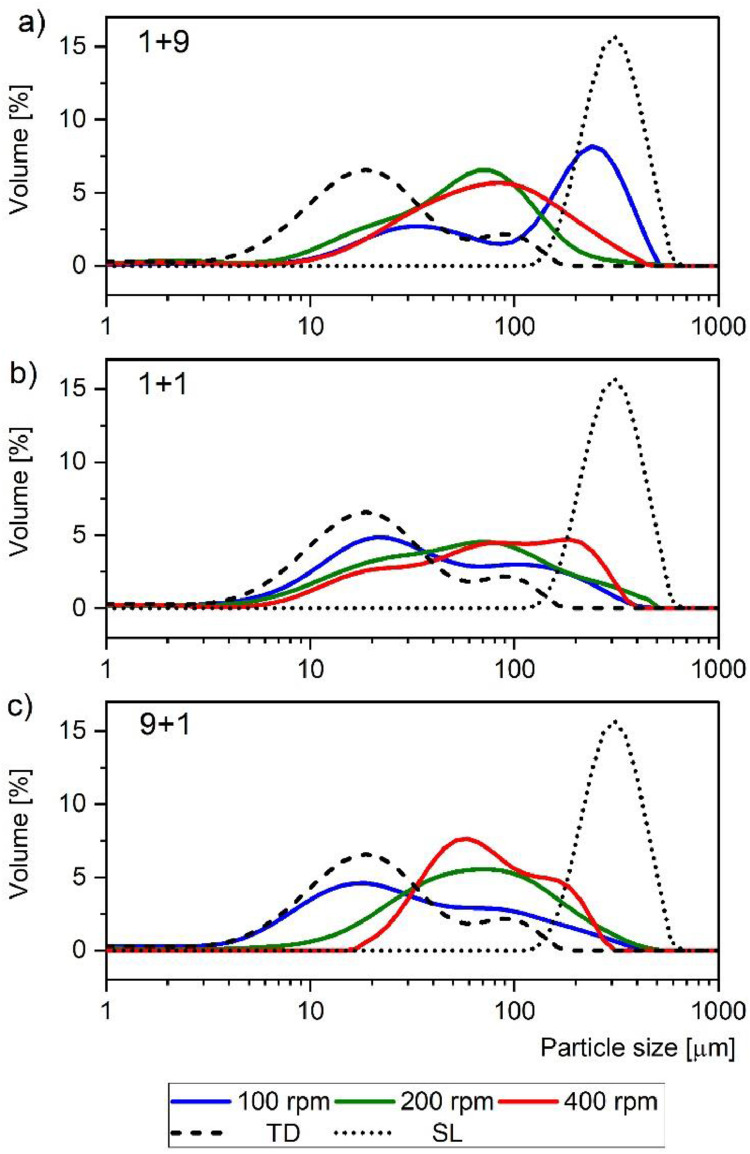
Impact of polymer load and rotational speed on particle size distribution of binary solid dispersions composed of tadalafil and Soluplus: (a) 10 % of tadalafil and 90 % (w/w) of Soluplus; (b) 50 % of tadalafil and 50 % (w/w) of Soluplus; (c) 90 % of tadalafil and 10 % (w/w) of Soluplus. Solid lines: blue – 100 rpm, green – 200 rpm, red – 400 rpm. Dash line crude tadalafil, dotted line crude Soluplus. Mean values of six measurements are presented.

The typical particle size distribution of the solid dispersions composed of tadalafil and Soluplus was acquired using a Mastersizer 3000 analyzer (Malvern Instruments Ltd., United Kingdom) equipped with a semiautomatic wet sample dispersion unit and a Hydro Sight dynamic imaging accessory. Data in the range from 0.01 µm to 3500 µm was collected with Mastersizer 3000 software. They were presented in Fig. 1 as frequency particle size distribution curves.

The impact of the formulation variable, that is, the polymer load on the properties of solid dispersions was investigated on three levels. Therefore, the frequency distribution curves, corresponding to the solid dispersions prepared in the tadalafil to Soluplus weight ratio 1+9, 1+1 and 9+1 (tadalafil+Soluplus) were shown in Fig. 1a, b, and c, respectively. In addition, the impact of the process-related variable, that is rotational speed of the solar disc in a planetary ball mill, on the performance of these solid dispersions was also assessed. This variable was tested on three levels as well, namely 100, 200, or 400 rpm. The results were presented in Fig. 1 in blue, green, or red solid lines, respectively. Regardless of formulation variables or processing conditions, all binary mixtures were milled for 1 h. For comparison reasons, the frequency distribution curves of crude tadalafil and crude Soluplus were also shown in Fig. 1.

All these frequency distribution curves (Fig. 1) represent mean values calculated from six measurements.

Raw data from Mastersizer 3000 software was exported to the MS 365 Excel, and saved in ASCII file format (*PSD*.csv). Each file contains data of six measurements and arithmetic mean calculated for these six samples with Mastersizer 3000 software. The first row contains name of the file, which encodes the solid dispersion composition, milling speed and date of measurement. The second row contains sample number (1-6). The third row includes column header. Next rows contain data of measurements, grouped in columns with particle size (µm) and volume (%).

**Fig. 2:**
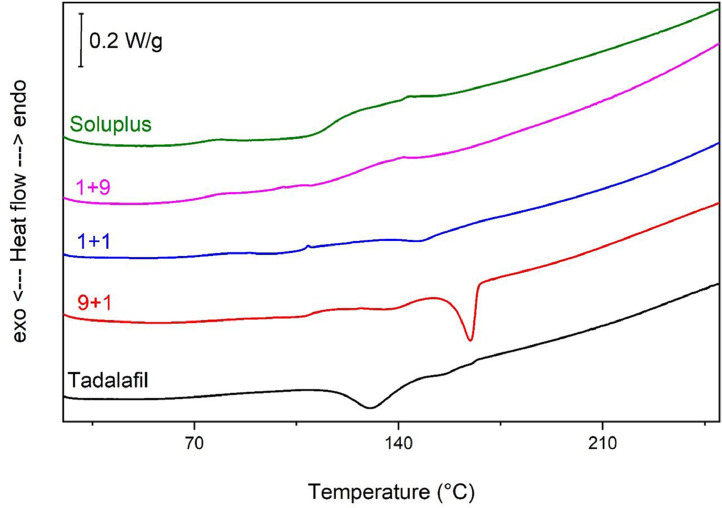
Heat flow curves recorded for (from top to bottom): crude Soluplus, solid dispersions composed of tadalafil and Soluplus co-milled in weight ratios 1+9, 1+1, 9+1 (tadalafil+Soluplus) for 1 h at 400 rpm, and fully amorphous tadalafil milled for 16 h at 400 rpm.

The thermal properties of ball-milled solid dispersions were examined with the aim of establishing the molecular interaction between a drug and a carrier induced by this co-processing [2,3]. Fig. 2 shows heat flow curves recorded for 1+9, 1+1 and 9+1 (tadalafil + Soluplus) solid dispersions ball milled for 1 h at 400 rpm. The heat flow curves typical of crude Soluplus and fully amorphous tadalafil prepared by high energy ball milling for 16 h at 400 rpm were presented as references (Fig. 2). All these heat flow curves were normalized to the sample weight and presented in function of temperature expressed in°C. Raw data was exported to ASCII format (DSC.txt)*.*

**Fig. 3:**
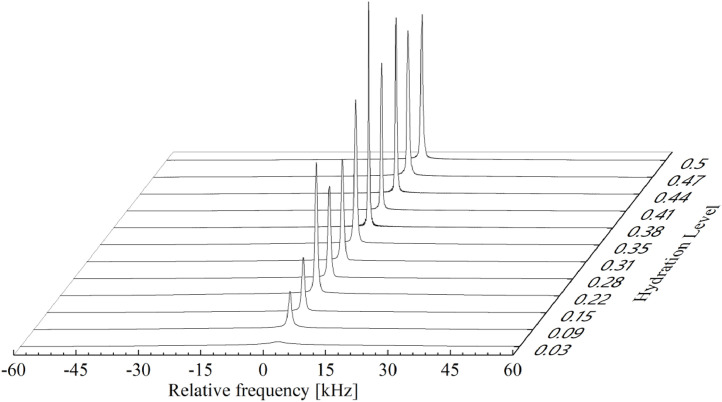
Raw ^1^H-NMR spectra of the hydration course of 1+9 solid dispersion milled at 200 rpm.

Raw Bruker TopSpin data, including all acquisition parameters, raw FIDs, etc. in order of ascending hydration levels was shared in the folder named *200_601_9_Raws* and further in folders (23 for 0.03, 29 for 0.09, etc. with rising order). ^1^H-NMR spectra with the obtained hydration levels were presented in Fig. 3.

**Fig. 4:**
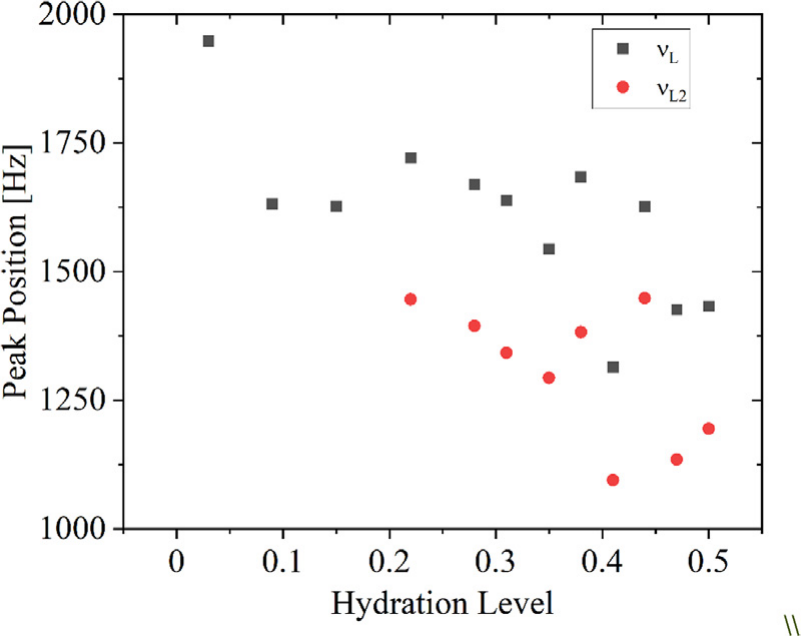
Peak positions of 1+9 solid dispersion (90% Soluplus, 200 rpm) hydration course: 1st Lorentzian function in black squares, 2nd Lorentzian function in red dots.

Data imported from Topspin spectra were presented in the *200_60_1_9* Origin project (OriginLab Pro 2021b, USA). Then, in Fig. 4 selected data was shown describing peak positions of the identified mobile protons fractions, which were attributed to the adsorbed water content in the solid dispersion.

## 2 Experimental Design, Materials and Methods

### 2.1 Preparation of Solid Dispersions

Tadalafil was combined with Soluplus in three different weight ratios (a formulation variable): 1+9, 1+1 and 9+1 [4,5]. The first number in the mixing ratio corresponds to tadalafl load while the second number corresponds to Soluplus load. These mixtures were high-energy ball milled for 1 h at room temperature, using a Pulverisette 7 classic planetary ball mill line (Fritsch, Idar-Oberstein, Germany). The samples of 1 g were placed in 45 mL grinding jars with seven milling balls of 1 cm in diameter. Both the jars and milling balls were made of zirconium dioxide. A ball-to-sample mass ratio was 75:1. The rotational speed of a solar disc was kept at three levels (a process variable): 100 rpm, 200 rpm, or 400 rpm.

### 2.2 Particle Size Distribution

The particle size was acquired with the Mastersizer 3000 laser diffraction particle size analyzer (Malvern Instruments Ltd., United Kingdom) equipped with the Hydro EV semi-automated wet sample dispersion unit and the Hydro Sight dynamic imaging accessory [6,7].

Due to the partial dissolution of the solid dispersions of tadalafil/Soluplus in water, the dispersing liquid used for the analysis was a cyclohexane (refractive index = 1.426). It was maintained at a temperature of 30.0 ± 0.1°C by a water jacket connected to an Accel 250LC (Thermo Fisher Scientific, USA) cooling/heating recirculating system. Cyclohexane was stirred with a constant speed of 1200 rpm throughout the entire analysis. The laser was aligned automatically and a background signal was recorded by the instrument software Mastersizer 3000 (v.3.60). The powdered samples were gradually added to the medium until the obscuration value was between 5% and 20%. Then the measurements were performed. Each sample was analyzed in six repetitions. Fraunhofer diffraction theory was used to find the particle size based on the light intensity distribution pattern. The average values were calculated using Mastersizer 3000 software. They were transferred to the project of OriginLab Pro 2021b software where the merged Fig. 1 was created.

### 2.3 DSC Analyses

DSC measurements were carried out as follows: the sample of ca. 5 mg was placed in an open 40 uL open pan (without the lid). It was heated to 60°C and stabilized at this temperature for 40 minutes. After this isothermal program, the samples were cooled from 60°C to 20°C. The cooling rate was 10°C/min. The samples were reheated from 20°C to 250°C with the heating rate of 5°C/min [8]. These measurements were carried out in an Ar atmosphere with gas flow of 60 mL/min. They were transferred to the project of OriginLab Pro 2021b software where Fig. 2 was created.

### 2.4 ^1^H NMR Measurements

Samples of solid dispersion (1+9) prepared by high-energy ball milling (at 200 rpm), were hydrated from the gaseous phase upon storage over the water surface [9,10]. The samples were introduced into NMR probes and placed in a desiccator. After acquiring an appropriate hydration level ± 1%, they were sealed with a paraffin film and ^1^H NMR spectra were recorded. A Bruker Avance III 300 spectrometer (Bruker Biospin) was used for aquisition, operating at the resonance frequency of 300 MHz for protons (at *B_0_* = 7 T) with a transmitter power equal to 400 W (*π/2 =* 1.5 μs, dead time 7.5 μs, repetition time 2 s) [11]. To aquaire data one *π/2* pulse where used and the Free Induction Decay was recorded. After averaging of 40 acquisitions and using Fourier transformation and phase optimalization at the end raw data where imported further. The data collected in TopSpin software were imported to the project of Origin (OriginLab 2021b, USA with ONMR add-on) where Figs. 3 and 4 were created. Superposition of 2 Gaussian and 1 or 2 Lorentzian functions (for hydration level above 0.22) where used to aquire the half-widths of the mobile protons to determine mobility of the protons. Peak positions (Fig. 4) can assess difference in chemical shift between mobile protons fractions. Data was exported into ASCII files (200_60_Raw.txt, 200_60_Summary.txt)

## Ethics Statements

Data in Brief's Guide for Authors contains detailed information on the ethical guidelines that all authors must comply with.

## CRediT authorship contribution statement

**Karol Kubat:** Conceptualization, Investigation, Data curation, Writing – original draft. **Anna Krupa:** Data curation, Writing – review & editing. **Witold Brniak:** Investigation, Funding acquisition, Data curation. **Agnieszka Węgrzyn:** Investigation, Funding acquisition, Data curation. **Dorota Majda:** Investigation, Funding acquisition, Data curation. **Agata Bogdał:** Funding acquisition. **Hubert Harańczyk:** Supervision.

## Declaration of Competing Interest

The authors declare that they have no known competing financial interests or personal relationships that could have appeared to influence the work reported in this paper.

## Data Availability

Tadalafil_Soluplus_Data (Original data) (Mendeley Data Repository).
